# Comparative study of the levonorgestrel intrauterine system and laparoscopic hysterectomy for the treatment of heavy menstrual bleeding in enlarged uteri

**DOI:** 10.31744/einstein_journal/2023AO0033

**Published:** 2023-03-24

**Authors:** Daniella De Batista Depes, Marcos Vinícius Maia da Mata, Ana Maria Gomes Pereira, João Alfredo Martins, Maíta Poli de Araújo, Reginaldo Guedes Coelho Lopes, Zsuzsanna Ilona Katalin de Jármy-Di Bella

**Affiliations:** 1 Instituto de Assistência Médica ao Servidor Público Estadual Hospital do Servidor Público Estadual “Francisco Morato de Oliveira” São Paulo SP Brazil Instituto de Assistência Médica ao Servidor Público Estadual, Hospital do Servidor Público Estadual “Francisco Morato de Oliveira”, São Paulo, SP, Brazil.; 2 Escola Paulista de Medicina Universidade Federal de São Paulo São Paulo SP Brazil Escola Paulista de Medicina, Universidade Federal de São Paulo, São Paulo, SP, Brazil.

**Keywords:** Menorrhagia, Uterine hemorrhage, Therapeutics, Hysterectomy, Intrauterine devices, Levonorgestrel, Laparoscopy

## Abstract

**Objective:**

To evaluate the effectiveness of the levonorgestrel intrauterine system in the treatment of patients with heavy menstrual bleeding and an enlarged uterus and to compare satisfaction and its complications with hysterectomy.

**Methods:**

This was a comparative cross-sectional observational study of women with heavy menstrual bleeding and an enlarged uterus. Sixty-two women were treated and followed up for four years. Insertion of the levonorgestrel intrauterine system was performed in Group 1, and laparoscopic hysterectomy was performed in Group 2.

**Results:**

In Group 1 (n=31), 21 patients (67.7%) showed improvement in the bleeding pattern, and 11 patients (35.5%) had amenorrhea. Five patients (16.1%) remained with heavy bleeding and were considered to have experienced treatment failure. There were seven expulsions (22.6%); in five patients, bleeding remained heavy, but in two patients the bleeding returned to that of normal menstruation. No relationship was found between treatment failure and greater hysterometries (p=0.40) or greater uterine volumes (p=0.50), whereas expulsion was greater in uteri with smaller hysterometries (p=0.04). There were 13 (21%) complications, seven (53.8%) in the group that underwent insertion of the levonorgestrel intrauterine system (all were device expulsions), and six (46.2%) in the surgical group, which were the most severe ones (p=0.76). Regarding satisfaction, 12 patients (38.7%) were dissatisfied with the levonorgestrel intrauterine system and one (3.23%) was dissatisfied with the surgical treatment (p=0.00).

**Conclusion:**

Treatment with the levonorgestrel intrauterine system in patients with heavy menstrual bleeding and an enlarged uterus was effective, and when compared with laparoscopic hysterectomy, it had a lower rate of satisfaction and the same rate of complications, although less severe.

## Highlights

• The levonorgestrel intrauterine system treatment prevents 67.7% of surgeries in patients with heavy menstrual bleeding and enlarged uteri.

**Figure:**
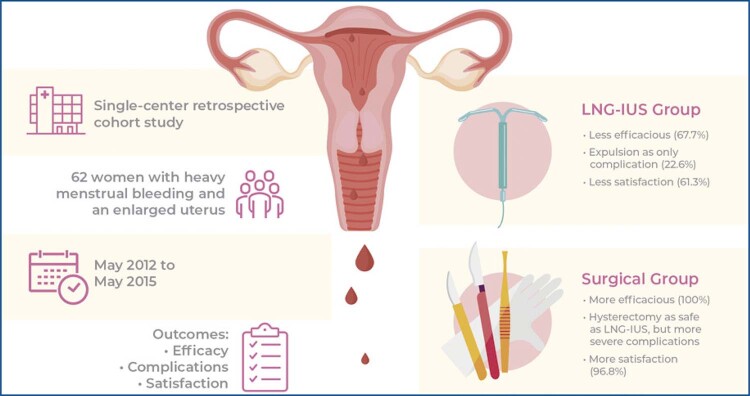


## INTRODUCTION

Heavy menstrual bleeding comprises a blood loss of 80mL or more per menstrual cycle, or blood loss that interferes with women’s physical, emotional, and social well-being, thereby reducing their quality of life.^( 1 , 2 )^It affects one-third of women of reproductive age and is the most common reason for gynecological consultation.^( 1 , 3 , 4 )^

Drug therapy is the initial approach, with the recommendation of hormonal and non-hormonal drugs; however, this treatment only results in a 40%-50% reduction in menstrual blood loss.^( 5 )^

Surgical treatment, on the other hand, includes all types of endometrial ablation and hysterectomy, the latter being the most commonly-performed procedure in Gynecology.^( 1 , 6 )^ The procedure is very effective but invasive, irreversible, and expensive.^( 7 , 8 )^ In the last 10-20 years, there has been a reduction in the number of hysterectomies performed worldwide.^( 6 )^

The levonorgestrel-releasing intrauterine system (LNG-IUS) is an effective alternative to heavy menstrual bleeding and has been increasingly used.^( 1 - 3 , 5 , 7 - 11 )^

In cases of heavy menstrual bleeding without a structural cause, there was a reduction of 86% after three months and 97% after 12 months.^( 5 , 12 )^

In 1999, a kidney transplant patient with multiple fibroids, treated with LNG-IUS due to contraindication to surgery, developed reduced bleeding, and paved the way for a new perspective of clinical treatment in these cases.^( 13 )^

Several authors have reported benefits with the use of LNG-IUS in patients with heavy menstrual bleeding due to structural causes; however, they reported a higher rate of device expulsion and treatment failure in patients with enlarged uteri.^( 14 - 16 )^

## OBJECTIVE

To evaluate the effectiveness of the levonorgestrel intrauterine system in the treatment of patients with heavy menstrual bleeding and enlarged uterus and to compare the satisfaction and complications of levonorgestrel intrauterine system with laparoscopic hysterectomy in the treatment of these patients.

## METHODS

This was a comparative retrospective cohort study performed at *Hospital do Servidor Público Estadual de São Paulo* with patients who were treated for heavy menstrual bleeding in an enlarged uterus between May 2012 and May 2015 through LNG-IUS insertion or laparoscopic hysterectomy (LH). This study was approved by the Research Ethics Committee of the *Universidade Federal de São Paulo* (CAAE: 80024117.5.3001.5505; # 3.031.115).

Consecutive patients seen at the Gynecology Outpatient Clinic refractory to clinical treatment (anti-inflammatory drugs, tranexamic acid, combined oral contraceptive pills, or progestogens) were included.

The inclusion criteria were as follows: menacme confirmed by regular cycles and/or follicle-stimulating hormone (FSH) doses <20mIU/mL; heavy menstrual bleeding (>3), and enlarged uterus with volume measured by pelvic endovaginal ultrasonography. Bleeding intensity was classified as: 0, absent (no bleeding); 1, menstrual spotting or leaks (presence of mild bleeding/use of sanitary pads only); 2, mild bleeding (less than the patient’s usual menstruation); 3, moderate bleeding (similar to the patient’s usual menstruation); and 4, heavy bleeding (greater than the patient’s usual menstruation).

In turn, an enlarged uterus was considered as that with a volume higher than expected for Brazilian women, with the average volume for this population being 90.48 cc and 105 cc in multiparous women.^( 17 )^

Patients with submucosal fibroids or endometrial polyps were excluded from the LNG-IUS Group treated with LNG-IUS.

Hence, two treatment options were made available for patients to choose: insertion of the LNG-IUS on an outpatient basis (Group 1) or surgery (Group 2), with total hysterectomy performed by LH as the standard procedure carried out in the service.

After an average of four years of intervention (between 31 and 74 months), the patients were invited to participate in an interview and signed an Informed Consent Form. The main evaluated factor was the effectiveness of the treatment, using the score on the bleeding intensity questionnaire and comparing the results obtained before and after the procedure. A Likert scale was used to assess patient satisfaction.^( 18 )^For the analysis of complications, all the information recorded in the medical record concerning the preoperative period and the immediate and late postoperative period was recorded, and the patient was verbally questioned about other possible complications until the evaluation appointment in the fourth year after treatment.

The degree of subjective satisfaction reported by the patients was classified according to the Likert scale, in five points: 1, totally dissatisfied; 2, little satisfied; 3, reasonably satisfied; 4, very satisfied; and 5, extremely satisfied.

The collected data were registered in an Excel spreadsheet for Windows^®^, Atlanta, GA, USA and analyzed using the Epi Info version 7 statistical program. Continuous variables are presented as means and standard deviations or as medians and quartiles, depending on distribution normality. Categorical variables are presented as percentages. Student’s *t* -test was used to compare means; and χ^2^and Fisher’s exact tests were used to compare frequencies between groups, considering a 95% confidence interval and p<0.05 was considered statistically significant.

## RESULTS

A total of 62 women were treated; most of them were black (53.2%), followed by white (45.2%) and Asian (1.6%). Regarding education, 54.8% had a higher level of education. Patients’ age ranged from 30 to 54 years, with a mean of 44.8 (±4.3) years in the LNG-IUS Group, and 44.9 (±4.8) years in the surgical group (p=0.90). The body mass index was 27 (±4.2) Kg/m^2^ in the LNG-IUS Group and 28 (±5.5) Kg/m^2^ in the Surgical Group (p=0.40) ( Table 1 ).

**Table 1:** Demographic and clinical data from Group 1 (levonorgestrel-releasing intrauterine system) and Group 2 (laparoscopic hysterectomy)

Demographic and clinical data	LNG-IUS n=31	Laparoscopic hysterectomy n=31	95%CI	p value
Age				
Mean±(SD)	44.8 (±4.3)	44.9 (±4.8)		0.90
BMI				
Mean±(SD)	27.2 (±4.2)	28.3 (±5.5)		0.40
Fibroids, n (%)	24 (77.4)	31 (100)	0.00-0.61	0.01*
Adenomyosis, n (%)	6 (19.4)	14 (45.2)	0.09-0.91	0.03*
Uterine volume				
Mean±(SD)	237.3 (±45.7)	244.9 (±78.4)		0.64

* significant.

SD: standard deviation; LNG-IUS: levonorgestrel-releasing intrauterine system; LH: laparoscopic hysterectomy; BMI: body mass index; 95%CI: 95% confidence interval.

Most had given birth up to two times: 71% in the LNG-IUS Group and 67.7% in the Surgical Group.

Of the total amount of patients, 25.8% had a definitive contraceptive method, and all reported heavy menstrual bleeding (>3). Regarding the cause of bleeding, 88.6% of the patients were diagnosed with uterine fibroids, 32.2% with adenomyosis, 14.4% with endometrial hyperplasia without atypia, and 1.6% with a non-structural cause. The uterine volume ranged from 141 cc to 423 cc, with a mean of 237.3 (±45.7) cc in Group 1, and 244.9 (±78.4) cc in Group 2 (p=0.64) ( Table 1 ). Hysterometry was performed in Group 1, ranging from 7 to 11cm, with a mean of 9.2 (±0.9) cm.

In Group 2, 18 hysterectomies were performed laparoscopically, and 13 underwent subtotal hysterectomies due to intraoperative technical difficulties. One patient (3.2%) experienced bleeding comparable to the usual menstrual volume after the procedure.

Table 2 compares the results of the treatments. LNG-IUS failed to reduce bleeding in 32.3% of patients, whereas hysterectomy was 100% effective (p=0.00). The dissatisfaction rate was higher in the LNG-IUS Group (38.7%) than in the Surgery Group (3.2%) (p=0.00).

**Table 2:** A comparison of the results obtained with the levonorgestrel-releasing intrauterine system and laparoscopic hysterectomy in the treatment of abnormal uterine bleeding

Parameter	LNG-IUS (n=31) n (%)	Laparoscopic hysterectomy (n=31) n (%)	OR (95%CI)	p value
Heavy bleeding (>3)	10 (32.3)	0 (0)	Undefined	0.00*
Complications	7 (22.6)	6 (19.4)	1.21 (0.36-4.14)	0.76
Dissatisfaction	12 (38.7)	1 (3.2)	18.95 (2.28-157.76)	0.00*

* significant.

OR: odds ratio; LNG-IUS: levonorgestrel-releasing intrauterine system; LH: laparoscopic hysterectomy; 95%CI: 95% confidence interval.

Regarding the response to treatment with LNG-IUS, 67.7% of the patients had a decreased bleeding pattern in relation to the initial one: 35.5% had amenorrhea, 12.9% reported leaks, 9.7% had bleeding less than normal menstruation, and 9.7% had bleeding similar to normal menstrual flow.

Thirteen patients had complications, six (19.4%) after hysterectomy and seven (22.6%) after LNG-IUS insertion (p=0.76). Postoperative complications included conversion to laparotomy, surgical wound dehiscence, vaginal vault granulation, infection with vaginal vault dehiscence, residual cervix bleeding, and bladder injury with the formation of vesicovaginal fistula. Seven expulsions occurred in the LNG-IUS Group ( Table 2 ).

The satisfaction rate >2 points (reasonably, very, or extremely satisfied) was 61.3% in Group 1 and 96.8% in Group 2 (p=0.00). All patients who failed to control their bleeding with LNG-IUS were dissatisfied ( Table 2 ).

Table 3 shows the possible relationships between LNG-IUS treatment failure and hysterometry, uterine volume, presence of fibroids, or adenomyosis. It was observed that uteri with the greatest hysterometries were not those that did not respond to treatment (p=0.40) or those with the highest volumes (p=0.50). Failure was unrelated to the presence of fibroids (p=0.73) or adenomyosis (p=0.27) ( Table 3 ).

**Table 3:** Analysis of characteristics of the group with treatment failure regarding the levonorgestrel-releasing intrauterine system

Variables	Bleeding <=3 (n=26) n (%)	Bleeding >3** (n=5) n (%)	OR (95%CI)	p value
Hysterometry >9cm	6 (60)	4 (40)	1.67 (0.34–8.09)	0.40
Uterus >237.3 cc	10 (83.3)	2 (16.7)	0.60 (0.08–4.45)	0.50
Fibroids	15 (79)	4 (21)	1.07 (0.10–12.4)	0.73
Adenomyosis	3 (60)	2 (40)	3.56 (0.40–31.2)	0.27

* excluded patients with eject device; ** Bleeding >3 is heavy, which characterizes treatment failure.

OR: odds ratio; 95%CI: 95% confidence interval.

In seven patients (22.6%), device expulsion occurred between 2 and 16 months after insertion; in two of them, the bleeding returned to that of a normal menstruation, and for five patients it returned to the pattern of a heavy flow.

For the seven expulsions, there was an inverse correlation with the quantitative hysterometry value. In uteri with hysterometry >9cm, there were no expulsions (p=0.40). There was no correlation between expulsion and uterine volume >237 cc (p=0.50), fibroids (p=0.73), or adenomyosis (p=0.27) ( Table 4 ). During subsequent evaluation, two of these patients with LNG-IUS expulsion were diagnosed with submucosal fibroids undetected on the initial ultrasound and two were diagnosed with endometrial hyperplasia on hysteroscopy, with images of pseudopolyps. The two patients with uteri >237 cc who expelled the device had intracavitary disorders.

**Table 4:** An analysis of characteristics of the group that presented expulsion of th e levonorgestrel-releasing intrauterine system

Variables	Without expulsion (n=24) n (%)	Expulsion (n=7) n (%)	OR (95%CI)	p value
Hysterometry >9cm	10 (100)	0 (0)	Undefined	0.04*
Uterus >237.3 cc	12 (85.7)	2 (14.3)	0.40 (0.65-2.48)	0.29
Fibroids	19 (79.2)	5 (20.8)	0.66 (0.10-4.46)	0.51
Adenomyosis	5 (83.3)	1 (16.7)	0.63 (0.06-6.54)	0.59

* significant.

OR: odds ratio; 95%CI: 95% confidence interval.

## DISCUSSION

Each year, 600.000 American women undergo hysterectomy, and by the age of 60 years, nearly one in three women will have undergone hysterectomy in the United States, with uterine fibroids being the greatest indication in general, due to heavy menstrual bleeding.^( 19 - 21 )^

Compared to LNG-IUS, hysterectomy provides a higher rate of satisfaction and effectiveness, although with more severe complications.^( 22 )^

A systematic review of 16 studies concluded that LNG-IUS is a cost-effective treatment for heavy menstrual bleeding without a structural cause, is well tolerated, and increases the quality of life when compared with medical and surgical treatments.^( 20 )^

Park et al. studied 48 patients with adenomyosis and enlarged uteri, comparable to 12 weeks of gestation or greater, and observed 68.8% success in replacing surgery with LNG-IUS, a value similar to that found in the present study, in which 67.7% of patients satisfactorily responded to treatment with LNG-IUS.^( 14 )^

Lee et al. conducted a retrospective study evaluating LNG-IUS in the treatment of patients with adenomyosis, and the only independent factor related to failure was uterine volume >150 cc.^( 15 )^

Conversely, Kim et al. reported that having a fibroid >2.5cm is a risk factor for treatment failure with LNG-IUS.^( 21 )^

The present study did not identify any isolated predisposing factors associated with LNG-IUS treatment failure. The five patients who did not respond to treatment had uteri with volumes between 169 and 252 cc.

Furthermore, according to Socolov et al., LNG-IUS does not seem to be a good method for patients with intracavitary fibroids because of the known risk of expulsion.^( 16 )^ Although our study excluded patients with intracavitary disorders, in the seven cases of expulsion, two patients had submucosal fibroids not diagnosed on the initial ultrasound, and two had, on hysteroscopy, endometrial hyperplasia with a polypoid aspect on hysteroscopy, which suggests that intracavitary disorders are the cause of greater expulsion of the device.

Park et al. observed 37.5% expulsion of the intrauterine device with levonorgestrel in enlarged uteri with adenomyosis in the first year of use, although the expulsion rate was not higher in the enlarged uteri.^( 14 )^In this study, the largest uterine cavity was a protective factor against device expulsion, and 71.4% of expulsions occurred in the first year after insertion.

The present study found a high rate of satisfaction in hysterectomized patients, which was higher than that in clinically treated patients. All surgeries were laparoscopic, which probably resulted in good surgical results.

Regarding the major complications of hysterectomy, Davies et al. reported a rate of 3% intraoperatively and 9% postoperatively. These rates have decreased with the advent of new and less invasive techniques.^( 1 )^In the present study, all surgeries were laparoscopic, with 19.4% of complications, and the bladder injury evolving to vesicovaginal fistula was the most severe.

Study limitations: as this was a retrospective study with a small number of participants, the results should be verified in other larger and prospective clinical studies.

## CONCLUSION

Treatment with a levonorgestrel-releasing intrauterine system was effective in patients with heavy menstrual bleeding and enlarged uteri. Compared with laparoscopic hysterectomy, the levonorgestrel-releasing intrauterine system had a lower satisfaction rate and the same rate of complications, although less severe.

Our study showed the possibility of reducing surgeries in the treatment of heavy menstrual bleeding in enlarged uteri. New studies are important to confirm our findings in addition to evaluating cost reduction.

## References

[1] Davies J, Kadir RA (2017). Heavy menstrual bleeding: an update on management. Thromb Res.

[2] Bitzer J, Heikinheimo O, Nelson AL, Caaf-Alsina J, Fraser IS (2015). Medical management of heavy menstrual bleeding: a comprehensive review of the literature. Obstet Gynecol Surv.

[3] Uhm S, Perriera L (2014). Hormonal contraception as treatment for heavy menstrual bleeding: a systematic review. Clin Obstet Gynecol.

[4] van Dijk MM, van Hanegem N, Lange ME, Timmermans A (2015). Treatment of women with an endometrial polyp and heavy menstrual bleeding: a levonorgestrel-releasing intrauterine device or hysteroscopic polypectomy?. J Minim Invasive Gynecol.

[5] Billow MR, El-Nashar SA (2016). Management of abnormal uterine bleeding with emphasis on alternatives to hysterectomy. Obstet Gynecol Clin North Am.

[6] Neis KJ, Zubke W, Fehr M, Römer T, Tamussino K, Nothacker M (2016). Hysterectomy for benign uterine disease. Dtsch Arztebl Int.

[7] Cozza G, Pinto A, Giovanale V, Bianchi P, Guarino A, Marziani R (2017). Comparative effectiveness and impact on health-related quality of life of hysterectomy vs. levonorgestrel intra-uterine system for abnormal uterine bleeding. Eur Rev Med Pharmacol Sci.

[8] Spencer JC, Louie M, Moulder JK, Ellis V, Schiff LD, Toubia T (2017). Cost-effectiveness of treatments for heavy menstrual bleeding. Am J Obstet Gynecol.

[9] Wouk N, Helton M (2019). Abnormal uterine bleeding in premenopausal women. Am Fam Physician.

[10] Nayar J, Nair SS, George NA (2018). Is LNG-IUS the one-stop answer to AUB?. J Obstet Gynaecol India.

[11] Miranda MT, Simó PA (2018). Aspectos éticos del uso del diu Mirena^®^en el tratamiento de la hemorragia menstrual severa. Cuad Bioét.

[12] Maybin JA, Critchley HO (2016). Medical management of heavy menstrual bleeding. Womens Health (Lond).

[13] Fong YF, Singh K (1999). Effect of the Levonorgestrel-Releasing Intrauterine System on Uterine Myomas in a Renal Transplant Patient. Contraception.

[14] Park DS, Kim ML, Song T, Yun BS, Kim MK, Jun HS (2015). Clinical experiences of the levonorgestrel-releasing intrauterine system in patients with large symptomatic adenomyosis. Taiwan J Obstet Gynecol.

[15] Lee KH, Kim JK, Lee MA, Ko YB, Yang JB, Kang BH (2016). Relationship between uterine volume and discontinuation of treatment with levonorgestrel-releasing intrauterine devices in patients with adenomyosis. Arch Gynecol Obstet.

[16] Socolov D, Blidaru I, Tamba B, Miron N, Boiculese L, Socolov R (2011). Levonorgestrel releasing-intrauterine system for the treatment of menorrhagia and/or frequent irregular uterine bleeding associated with uterine leiomyoma. Eur J Contracept Reprod Health Care.

[17] Mauad F, Beduschi AF, Meschino RA, Mauad FM, Casanova MS, Ferreira AC (2001). Avaliação ultra-sonográfica das variações do volume uterino. Rev Bras Ginecol Obstet.

[18] Likert R (1932). A technique for the measurement of attitudes. Arch Psychology.

[19] Gunes M, Ozdegirmenci O, Kayikcioglu F, Haberal A, Kaplan M (2008). The effect of levonorgestrel intrauterine system on uterine myomas: a 1-year follow-up study. J Minim Invasive Gynecol.

[20] Health Quality Ontario (2016). Levonorgestrel-Releasing Intrauterine System (52 mg) for Idiopathic Heavy Menstrual Bleeding: a Health Technology Assessment. Ont Health Technol Assess Ser.

[21] Kim JY, No JH, Kim K, Kim YB, Jee BC, Lee JR (2013). Effect of myoma size on failure of thermal balloon ablation or levonorgestrel releasing intrauterine system treatment in women with menorrhagia. Obstet Gynecol Sci.

[22] Louie M, Spencer J, Wheeler S, Ellis V, Toubia T, Schiff LD (2017). Comparison of the levonorgestrel-releasing intrauterine system, hysterectomy, and endometrial ablation for heavy menstrual bleeding in a decision analysis model. Int J Gynaecol Obstet.

